# Fabrication of Wearable PDMS Device for Rapid Detection of Nucleic Acids via Recombinase Polymerase Amplification Operated by Human Body Heat

**DOI:** 10.3390/bios12020072

**Published:** 2022-01-27

**Authors:** Kieu The Loan Trinh, Nae Yoon Lee

**Affiliations:** 1 Department of Industrial Environmental Engineering, Gachon University, 1342 Seongnam-daero, Sujeong-gu, Seongnam-si 13120, Gyeonggi-do, Korea; tktloan@gmail.com; 2 Department of BioNano Technology, Gachon University, 1342 Seongnam-daero, Sujeong-gu, Seongnam-si 13120, Gyeonggi-do, Korea

**Keywords:** wearable RPA microdevice, poly(dimethylsiloxane) (PDMS), human body heat, recombinase polymerase amplification (RPA)

## Abstract

Pathogen detection by nucleic acid amplification proved its significance during the current coronavirus disease 2019 (COVID-19) pandemic. The emergence of recombinase polymerase amplification (RPA) has enabled nucleic acid amplification in limited-resource conditions owing to the low operating temperatures around the human body. In this study, we fabricated a wearable RPA microdevice using poly(dimethylsiloxane) (PDMS), which can form soft—but tight—contact with human skin without external support during the body-heat-based reaction process. In particular, the curing agent ratio of PDMS was tuned to improve the flexibility and adhesion of the device for better contact with human skin, as well as to temporally bond the microdevice without requiring further surface modification steps. For PDMS characterization, water contact angle measurements and tests for flexibility, stretchability, bond strength, comfortability, and bendability were conducted to confirm the surface properties of the different mixing ratios of PDMS. By using human body heat, the wearable RPA microdevices were successfully applied to amplify 210 bp from *Escherichia coli* O157:H7 (*E. coli* O157:H7) and 203 bp from the DNA plasmid SARS-CoV-2 within 23 min. The limit of detection (LOD) was approximately 500 pg/reaction for genomic DNA template (*E. coli* O157:H7), and 600 fg/reaction for plasmid DNA template (SARS-CoV-2), based on gel electrophoresis. The wearable RPA microdevice could have a high impact on DNA amplification in instrument-free and resource-limited settings.

## 1. Introduction

Nucleic acid amplification testing has become the gold standard diagnostic method applied in various fields, such as pathogen detection, early cancer diagnosis, and forensic identification [1,2]. Polymerase chain reaction (PCR) is the most common technique for amplifying nucleic acids and has been proven to be an accurate method, with high specificity and sensitivity [3,4,5]. Once again, during the coronavirus disease 2019 (COVID-19) pandemic, PCR has proved to be the most reliable method for the early, rapid detection of COVID-19 [6,7,8]. However, PCR usually requires several hours for the amplification process using a thermal cycler, which makes it difficult to utilize its applications in resource-limited settings where the pandemic and burden of many infectious diseases are the most severe [9].

Alternative methods include isothermal amplification, such as loop-mediated isothermal amplification (LAMP) [10,11], helicase-dependent amplification (HDA) [12,13], and recombinase polymerase amplification (RPA) [14,15,16,17]. Among the various isothermal amplification methods, RPA, which was introduced in 2006, has been applied to amplify nucleic acids because of its high sensitivity and rapid amplification at low temperatures (30–40 °C), which is a relatively similar temperature range to that of the human body [18,19]. The mild-acting temperature of RPA allows simple operation by skin contact and eliminates the need for an external heating device. Because of this benefit, RPA can be an ideal choice for building a point-of-care (POC) device for nucleic acid amplification under low-resource setting conditions. Several studies have utilized this characteristic in developing RPA devices that work at human body temperature [20,21]. These devices were held tightly to the skin using external supports, such as adhesive bandages or rubber bands, since tight contact with the skin is necessary for efficient heat transfer from the skin to the reaction solution.

Poly(dimethylsiloxane) (PDMS) is a silicone-based elastomer that is widely used in a variety of analytical fields owing to its flexibility, transparency, and biocompatibility [22,23]. In the field of wearable devices, PDMS is one of the most popular choices of materials because it can provide conformal contact with different surfaces and be easily wrapped around curvatures due to its low Young’s modulus (<2 MPa) [24,25,26]. In addition, the assembly of PDMS devices is normally conducted by treating oxygen plasma or corona discharge to achieve permanent bonding [27]. Although this bonding method can support PDMS devices against harsh reaction conditions or distortion, an oxygen plasma machine is expensive and corona treatment over a large area is difficult. However, in applications that use mild working conditions, such as cell culturing, PDMS devices assembled without any surface treatment were able to hold tightly without any leakage [28,29]. Therefore, an RPA wearable device that works at body temperature eliminates the need for an expensive oxidation process for permanent bonding. In addition, PDMS can be tailored to improve its flexibility and adhesion for more stable contact between PDMS and PDMS and PDMS-skin by simply tuning the curing agent ratio [30].

In this study, a simple method for fabricating a wearable microdevice was introduced using flexible soft-contact PDMS to amplify nucleic acids by RPA. The PDMS was mixed with different ratios of pre-polymer and curing agent to achieve high stretchability and flexibility without changing the basic characteristics of PDMS. Water contact angle measurements and bonding performance tests with glass were performed to prove that the basic properties of PDMS remained, even with the change in curing agent ratio. Next, PDMS was used as a material for fabricating a wearable microdevice based on soft lithography and the two PDMS substrates were bonded without requiring any extra surface modification. Furthermore, a leak test was conducted to demonstrate a leak-free microdevice at room temperature. For applications, the fabricated wearable microdevice was used to amplify the DNA fragments from gDNA of a major foodborne pathogen [*Escherichia coli* O157:H7 (*E. coli* O157:H7)] and a plasmid vector of SARS-CoV-2 from COVID-19 using RPA assays.

## 2. Materials and Methods

### 2.1. Materials

TwistAmp Basic kits were purchased from TwistDx (Cambridge, UK). Poly(dimethylsiloxane) (PDMS) pre-polymer (Sylgard 184) and curing agent were purchased from Dow Corning (Midland, MI, USA). *Taq* DNA polymerase, PCR buffer solutions, and dNTPs were purchased from BioFact (Daejeon, Korea). Primer pairs were purchased from Bioneer (Daejeon, Korea). The DNA ladder (100 bp) was purchased from Takara (Shiga, Japan) and agarose powder was purchased from BioShop (Ontaria, Canada). Loading Star was purchased from DYNE Bio (Seongnam, Korea). SYBR Green I (10,000×) was purchased from Lonza (Basel, Switzerland). Luria Bertani (LB) broth (with low salt) and agar powder (bacteriological) were purchased from MB cells (Seoul, Korea).

### 2.2. Production of the Flexible PDMS with Different Mixing Ratios

To produce PDMS with high flexibility as well as soft contact, mixtures with ratios of 10:1, 10:0.8, 10:0.6, and 10:0.4 (*w*/*w*, pre-polymer to curing agent) were prepared. The PDMS mixtures with different ratios were cast inside Petri dishes and cured for 2 h at 80 °C. To characterize each PDMS, the water contact angles, bonding performance, and flexibility (twisting, softness, and adhesiveness) of each sample were evaluated. A Phoenix 300 contact angle analyzer (Surface Electro Optics, Suwon-si, Gyeonggi-do, Korea) was used to measure water contact angle at the Core Facility for Bionano Materials at Gachon University. A texture analyzer (QTS 25, Brookfield, Middleboro, MA, USA) was used to evaluate the strain and bond strength. For the strain, PDMS substrates (4 cm in length, 1 cm in width, and 600 μm in thickness) were held in two grips, and the loading arm moves up at a constant speed (100 mm/min) to achieve the “dogbone” shape of PDMS substrates. For bond strength measurement, twine was embedded into the PDMS pre-polymers and cured. After attaching two PDMS substrates, the twines were pulled apart at a speed of 100 mm/min. Each sample was measured three times, on the same day.

### 2.3. Design and Fabrication of Wearable Microdevice

**Figure 1:**
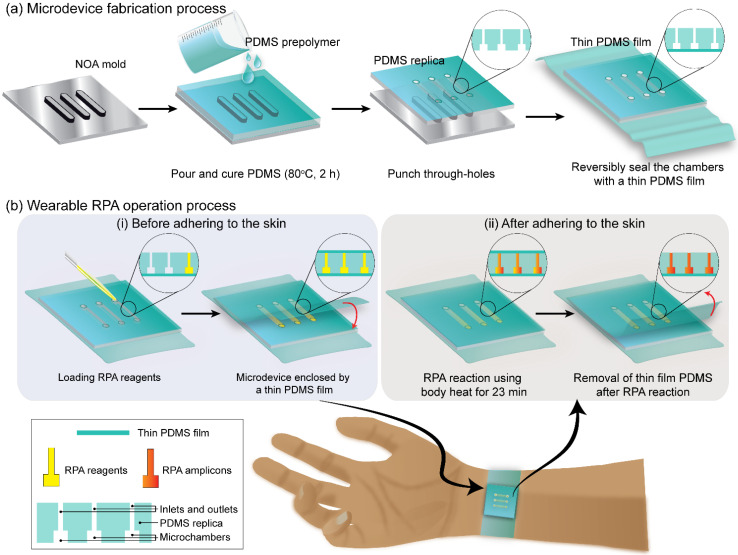
(**a**) Schematic illustration showing the fabrication of a wearable PDMS microdevice. (**b**) Schematic illustration showing the operation of a wearable PDMS microdevice.

Figure 1 shows the overall schematic for fabricating and operating a wearable PDMS microdevice. The wearable PDMS microdevice was fabricated using standard replica molding processes to create microchambers containing RPA reagents as shown in Figure 1a. Briefly, the Norland optical adhesive mold (NOA mold) of the microchambers was fabricated on a PET film using a soft lithography method. Next, the pre-polymer PDMS was prepared by mixing the pre-polymer and curing reagents at 10:0.4 (*w/w*) ratios by weight and poured onto the NOA mold. After 2 h of incubation at 80 °C, the cured replica PDMS was peeled off and the inlet and outlet were made using a hole puncher (1 mm in dimension). In addition, a flat PDMS thin film (1 mm in thickness) was fabricated using the same conditions as the replica PDMS. The patterned side of the PDMS replica was then aligned and placed in contact with the PDMS thin film to create a reversible bond due to the increased soft-contact and friction of PDMS, which did not require oxygen plasma treatment. As a result, the flexible wearable PDMS microdevice was completed, and the PDMS thin film was placed on the human skin to perform the RPA assays. In addition, Figure 1b shows the schematic illustration of a wearable RPA microdevice operation process. In brief, there were two steps including RPA reagent loading and RPA reaction performed before and after adhering the wearable microdevice to the human skin. As shown in Figure 1(bi), the microdevice was covered by another thin PDMS film for enclosing the microdevice chamber after loading the RPA reagents. This avoids the sample loss while the volunteers are freely moving during the RPA reaction. After the reaction, the microdevice was removed to analyze the RPA amplicons (Figure 1(bii)). Steps of operation could be flexibly changed depending on the position of the adhered microdevice for the comfort of volunteers. 

### 2.4. Sample Preparation and DNA Extraction

In this study, a foodborne pathogen (*E. coli* O157:H7 (ATCC 43895)) and one clinical virus (COVID-19) were tested. *E. coli* O157:H7 bacteria were grown in both liquid culture media and agar plates. *E. coli* O157:H7 was cultured in 5 mL of LB broth (10 g of tryptone, 5 g of yeast extract, and 5 g of NaCl in 1 L of distilled water) at 37 °C for 16 h, with constant shaking at 200 rpm. Viable counts were determined by performing serial dilution plating on solid LB agar media for *E. coli* O157:H7 and incubating at 37 °C for 16 h. For DNA extraction from *E. coli* O157:H7, the bacterial DNA was extracted from a solution that was cultured overnight via the Wizard Genomic DNA purification kit. The extracted gDNA was stored at 4 °C for short-term storage. In addition, with the ongoing global pandemic of COVID-19, a commercially available SARS-CoV-2 plasmid DNA was employed as a template to amplify the DNA for potential clinical applications.

### 2.5. Temperature Measurement

In this study, human body heat was used to conduct all of the RPA assays. Human body heat was measured using a non-contact precision infrared thermometer (Daihan Scientific Co., Ansung-si, Gyeonggi, Korea). In the comparison, an IR camera was used to measure the human body heat as a control experiment. In this way, 1 mm thin PDMS substrates were attached to the human body at four positions, as described previously. To evaluate the temperature distribution of human skin, 10 spots were randomly selected from each position of the chest, forearm, wrist, and fist for a 30 min period. 

### 2.6. Nucleic Acid Amplification by RPA

The primer sequences used to amplify 210 bp of the Shiga toxin gene in *E. coli* O157:H7 were designed as follows: 5′-TGT AAC TGG AAA GGT GGA GTA TAC A–3′ (forward) and 5′-GCT ATT CTG AGT CAA CGA AAA ATA AC–3′ (reverse) [31]. The primer sequences for amplifying a 203 bp gene fragment in SARS-CoV-2 plasmid DNA were as follows: 5′–ACT CTT TCG TGA GCG AGG AA–3′ (forward) and 5′–CCT GGA GCT GTT CAG GTT CT–3′ (reverse). A SARS-CoV-2 plasmid DNA (100 ng/μL) was further diluted and added to the RPA reagent to obtain a final template concentration of 600 fg/reaction. The extracted DNA of *E. coli* O157:H7 (240 ng/μL) was further diluted and added to the RPA reagent to achieve a final template concentration of 500 pg/reaction. To perform RPA, on-tube RPA was demonstrated and optimized according to the instructions provided in the TwistAmp Basic kits. The detailed schematics for RPA for DNA amplification were illustrated as shown in Figure S1. Briefly, the RPAs were performed using a 15 μL sample containing a 2× reaction buffer, 0.64 mM dNTP mixture, 10× Basic E-mix, and 10 μM of forward and reverse primers. All RPA reactions were incubated for 23 min using human body heat for all experiments. For control experiments, conventional PCR assays were performed on a thermal cycler (Bio-Rad C1000 thermal cycler) to compare the sensitivity and specificity of the RPA reactions. The PCRs were performed using a 25 μL sample containing a 5 μL buffer, 0.16 nM dNTPs mixture, 0.5 μM forward and reverse primers and 0.5 U/μL of *Taq* DNA polymerase. The input DNA of SARS-CoV-2 plasmid DNA and extracted DNA of *E. coli* O157:H7 were prepared at the same concentration in the RPA assays. The PCR amplifications were performed for 32 thermal cycles at 95 °C for 30 s for denaturation, 54 °C for 30 s for annealing, and 72 °C for 30 s for elongation, with an initial denaturation at 95 °C for 3 min, followed by a final extension step at 72 °C for 5 min. The amplicons were confirmed via gel electrophoresis on a 1.5% agarose gel. For rapid visual amplification, 0.1 μL of SYBR Green I (10×) was added to the RPA samples to realize the on-site detection of amplicons using an inverted fluorescence microscope (Olympus IX71) and the fluorescence signals were recorded and analyzed using ProgRes Capture Pro 2.8 software (Jenoptik). The intensities of RPA and PCR target bands from the gel images were further evaluated using ImageJ software. 

## 3. Results

### 3.1. Water Contact Angle Measurement

**Figure 2:**
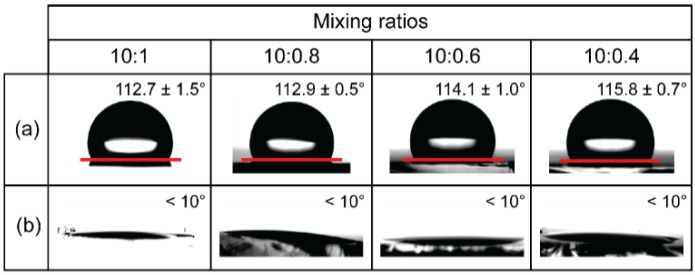
Characterization of PDMS substrates with different mixing ratios. Results of water contact angles measurements on PDMS substrates: (**a**) Pristine PDMS. (**b**) Oxygen plasma treatment of the PDMS. Five measurements were taken and averaged.

As shown in Figure 2, contact angle measurements were performed to evaluate the effect of the mixing ratio of the PDMS pre-polymer and curing agent on the PDMS surface properties. The measured contact angles of PDMS with different mixing ratios (10:1, 10:0.8, 10:0.6, and 10:0.4 (*w*/*w*)) were approximately 112.7 ± 1.5°, 112.9 ± 0.5°, 114.7 ± 1.0°, and 115.8 ± 0.7°, respectively. As shown in Figure 2b, after the oxygen plasma treatment, all contact angles dropped to less than 10°, indicating the hydrophilic stage of the PDMS surfaces. Based on the results shown in Figure 2a,b, we can conclude that the innate hydrophobicity of PDMS was not altered due to the reduced level of the curing agent.

### 3.2. Characterization of the PDMS Substrates with Different Mixing Ratios

**Figure 3:**
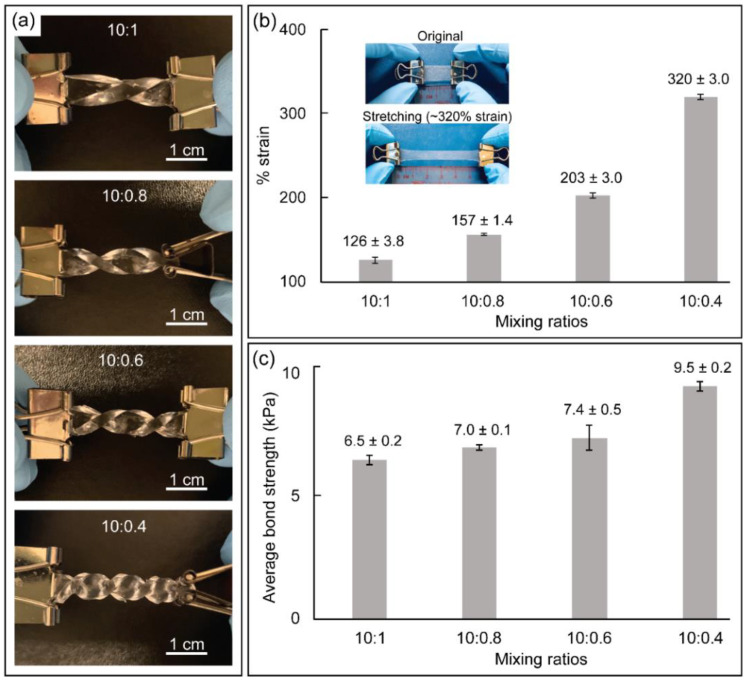
(**a**) Twisting test showing the flexibility of the PDMS fabricated with different mixing ratios of the pre-polymer and a curing agent. (**b**) Strain resolution of the PDMS substrate with different mixing ratios. The inset shows the photographs of the 10:0.4 (*w/w*) mixing ratio of PDMS substrate at different strains (original and stretching conditions). (**c**) Results of bond strength performed on PDMS–PDMS with different mixing ratios. All experiments were performed in triplicate.

To utilize PDMS for wearable device fabrication, a series of key performance indicators, including flexibility, stretchability, bond strength, comfortability, and bendability, were measured and characterized. First, a flexibility test was conducted to demonstrate that PDMS can be twisted, displaying flexible properties [32]. As shown in Figure 3a, the 2 mm thick PDMS with mixing ratios of 10:1, 10:0.8, 10:0.6, and 10:0.4 (*w/w*) allowed for twisting at 360°, 540°, 720°, and 1260°, respectively, and did not deform the PDMS. As shown in Figure 3b, 600 μm thick PDMS with a mixing ratio of 10:1, 10:0.8, 10:0.6, and 10:0.4 (*w/w*) were gradually pulled until the maximum stretch. The 10:1 PDMS was only able to stretch slightly; however, with decreasing curing agent ratio, the strain percentage significantly increased to values of 126 ± 3.8%, 157 ± 1.4%, 203 ± 3.0%, and 320 ± 3.0% for 10:1, 10:0.8, 10:0.6, and 10:0.4 (*w/w*), respectively. The inset shows photographs of the PDMS substrate with a 10:0.4 (*w/w*) mixing ratio before and after stretching. These results show that the 10:0.4 PDMS substrate is highly suitable for wearable devices owing to its highly flexible and stretchable nature. As shown in Figure 3c, the bond strengths of the PDMS–PDMS bonding with mixing ratios of 10:1, 10:0.8, 10:0.6, and 10:0.4 (*w/w*) were measured to be approximately 6.5 ± 0.2 kPa, 7.0 ± 0.1 kPa, 7.4 ± 0.5 kPa, and 9.5 ± 0.2 kPa, respectively. It is worth noting that two PDMSs adhered to each other without requiring surface modification. As a result, the 10:0.4 (*w/w*) mixing ratio of PDMS achieved the highest bond strength among the other mixing ratios. In addition, the 10:0.4 mixing ratio of PDMS was further employed for comfortability, bendability, and leak tests, as shown in Figure S2. As shown in Figure S2, the PDMS mixing ratio of 10:0.4 (*w*/*w*) also demonstrates a high softness and adhesiveness due to its high friction with human skin or other materials, as per previous studies [26]. The wearable microdevice was firmly attached to the human forearm without external support and the contact between the wearable microdevice and human skin was maintained, even when the device was turned upside down, as shown in Figure S2a. Therefore, the microdevice could withstand the random movement of the wearers. Furthermore, Figure S2c shows the results of the static leak test performed on the flexible wearable microdevice. Although non-plasma bonding of PDMS was applied to fabricate the wearable microdevice, it could still prevent the ink solution from leaking for over 96 h at room temperature. The results demonstrate that the lower curing agent ratio results in excellent flexibility for using PDMS in various wearable microdevices-related applications. For all subsequent experiments, a 10:0.4 (*w*/*w*) mixing ratio of PDMS was used owing to its incredible flexibility, which satisfies the requirements of a wearable RPA microdevice.

### 3.3. Sensitivity Analysis of the RPA Assays Using Human Body Heat for the Detection of E. coli O157:H7

**Figure 4:**
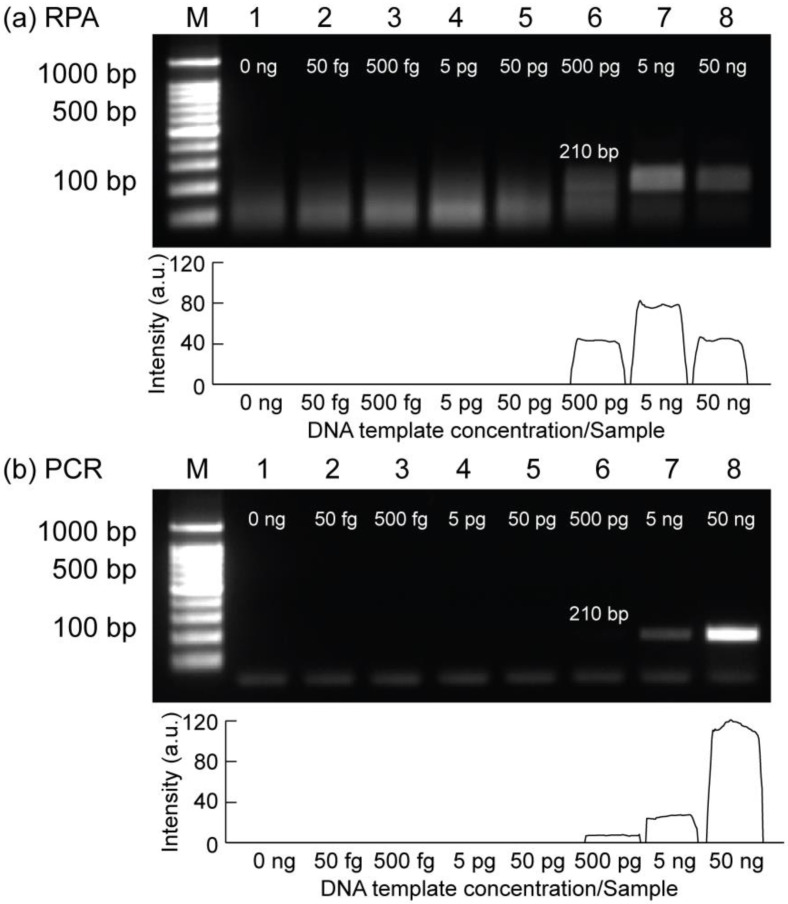
Results of gel electrophoresis showing the sensitivity using tenfold serial dilutions of gDNA input from *E. coli* O157:H7 bacteria. (**a**) On-tube RPA results. (**b**) On-tube PCR results. Lane M is the 100 bp DNA ladder. Lanes 1–8 show the amplification results when input gDNA concentrations were 50 fg to 50 ng/sample, respectively. Relative intensity scales of the target amplicons were shown below each gel image. All the experiments were repeated three times.

The sensitivity test is one of the most important criteria for the early detection of bacterial pathogens based on DNA amplification. Therefore, the sensitivity of RPA was evaluated using tenfold serial dilutions of the extracted gDNA from *E. coli* O157:H7 bacteria, as shown in Figure 4a. As shown in the gel image, lanes 1–8 represent from 50 fg to 50 ng/reaction. From the results, RPA successfully amplified the 210 bp target gene from genomic DNA when the input DNA concentration was from 500 pg to 50 ng/reaction. Accordingly, the limit of detection (LOD) of RPA was 500 pg/reaction within 23 min of incubation using human body heat. In addition, by using the same amount of input genomic DNA, the LOD of PCR was also conducted to compare with the LOD of RPA assays, as shown in Figure 4b. Similarly, the results indicated that PCR and RPA shared the same sensitivity value (500 pg/reaction) based on gel electrophoresis. However, in the case of PCR, the target band intensity signal was relatively weak when the input DNA was 500 pg/reaction, compared with RPA results. It can be concluded that the LOD of RPA can achieve a similar sensitivity for the same amount of input DNA as PCR while having the advantages of easier access and operation for the amplification reaction using point-of-care testings (POCTs). Furthermore, 500 pg (genomic DNA) was considered as a limit of detection for detecting *E. coli* O157:H7 using both RPA and PCR assays, and this concentration value was used for all subsequent on-tube and on-chip RPA experiments.

### 3.4. Results of on-Chip RPA Performed Using Human Body Heat for the Detection of E. coli O157:H7

**Figure 5:**
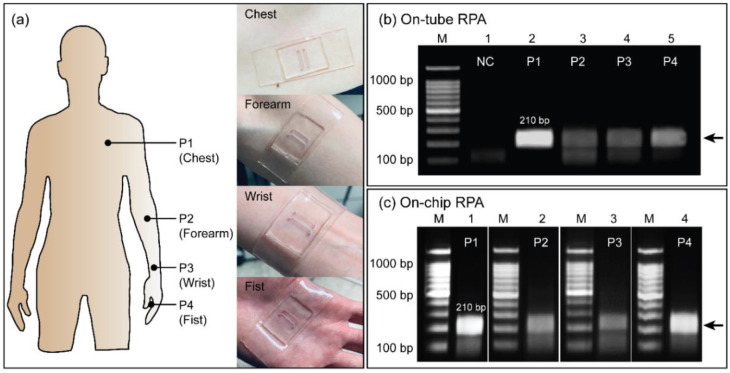
Wearable PDMS microdevice for RPA assays was performed using human body heat. (**a**) Illustration of four different positions (P1—chest; P2—forearm; P3—wrist; and P4—fist) and real photographs of the wearable PDMS microdevice adhered on a volunteer’s skin which was utilized to perform RPA using human body heat. (**b**) Agarose gel electrophoresis of tube-based RPA assays using human body heat at four different positions (P1 to P4). (**c**) Agarose gel electrophoresis of the wearable PDMS microdevice-based RPA assays using human body heat at four different positions (P1 to P4).

Figure 5 shows the RPA results from using human body heat to amplify *E. coli* O157:H7. The soft-contact wearable PDMS microdevices formed tight contact with human skin at most positions, such as the chest (P1), forearm (P2), wrist (P3), and fist (P4), as shown in Figure 5a. It was clearly shown that the wearable PDMS microdevice directly adhered to the skin without the use of any support bandages or tapes because of its soft-contact and flexibility. From this result, the wearable PDMS microdevice can maintain a conformal contact with the skin during the RPA reaction, reducing heat loss and improving heat transfer to achieve a homogenous heat profile. Before performing RPA using human body heat, RPAs were first performed on thermocycler with a temperature ranging from 30–40 °C as shown in Figure S3a. Afterward, RPA was performed on three volunteers with slightly different body temperatures (Figure S3b). Interestingly, all RPAs were successfully performed with negligible difference in the target intensities. From these results, we can conclude that the RPA can take place under normal body heat even with slight variations from human to human. Although the leak test result strongly supports the robustness of the sealing between the two pieces of PDMS over 96 h at room temperature (Figure S2c), it is worth noting that the wearable RPA device is sealed by reversible bonding. Therefore, it would be better to minimize movements such as forceful pushing or pulling during the RPA amplification to avoid any chances of leakage, which may cause cross-contamination or a false-positive result. Moreover, for high-risk DNA samples, the wearable RPA microdevice can be irreversibly sealed using the oxygen plasma bonding method; it would be a more preferred and safer approach for practical application. To demonstrate the applicability of this device, RPAs were conducted by amplifying the 210 bp target gene from *E. coli* O157:H7 using human body heat at four positions, as mentioned previously. Before performing on-chip RPA amplification, on-tube RPAs were first performed using a 0.5 mL tube to evaluate the effect on the amplification efficiency of human body heat at different positions, as shown in Figure 5b. The tube was attached to the human skin for RPA via a commercially available adhesive tape, as shown in Figure S4. As shown in the gel image, RPAs successfully amplified the 210 bp target gene using human body heat from the P1 to P4 positions (lanes 2–5). The target band was clearly expressed as a single band, indicating the specificity of the amplification, whereas no band was observed in the negative control (lane 1). The intensities of the RPA products obtained from P2 and P3 positions were approximately 53.2% of those obtained from P1 and P4, still demonstrating distinguishable amplicons, probably due to its higher heat distribution and stable temperature during RPA assays, as shown in Figures S5 and S6. The RPA using body heat was further tested on the microdevice, as shown in Figure 5c. The 210 bp target band was successfully amplified in the chambers within 23 min of incubation at the four positions (P1–P4). Once again, P1 and P4 (lanes 1 and 4) exhibited higher and clearer amplification results of the target band than those obtained from P2 and P3 (lanes 2 and 3), which corresponded with the on-tube results, as shown in Figure 5b. The intensities were approximately 2-fold higher (57.9%) for the results obtained from P1 and P4 compared to those obtained from P2 and P3. Therefore, the on-chip RPA results showed an almost similar trend to those obtained from the on-tube RPA. From these results, the fabricated wearable microdevice can be applied for DNA amplification through RPA assays using human body heat from various positions. Among the four positions, the forearm was selected to perform all RPA assays for easier access and a comfortable operating position.

### 3.5. Amplification of Plasmid DNA of COVID-19 Using the Wearable RPA Device

**Figure 6:**
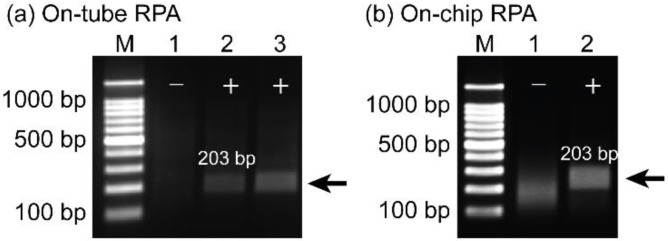
(**a**) On-tube RPA results of a 203 bp target gene using plasmid DNA of SARS-CoV-2. (**b**) On-chip RPA results of a 203 bp target gene from plasmid DNA of SARS-CoV-2. Lane M is the 100 bp DNA ladder. Lane 1 is a negative sample. Lanes 2 and 3 are the positive samples. All the experiments were repeated three times.

In addition to foodborne pathogens, the wearable RPA microdevice was also applied to amplify the 203 bp gene fragment from the plasmid DNA of SARS-CoV-2. Before performing on-chip RPA, the LOD of RPA was determined to be approximately 600 fg/reaction, as shown in Figure S7a. Based on this result, 600 fg were added into RPA reagents for all the following RPA assays. As shown in Figure 6, after 23 min of incubation, the 203 bp target gene was successfully amplified on the tube (Figure 6a) and wearable microdevice (Figure 6b), as confirmed by gel electrophoresis. In both gel images, lane 1 shows a negative control without the target band. The target band was displayed on a gel image with high specificity and selectivity (lanes 2 and 3 for on-tube RPA; lane 2 for on-chip RPA). In addition, PCR was also utilized to confirm the specificity and sensitivity of RPAs for amplifying the 203 bp target gene by using the same amount of input DNA, and the LOD was approximately 60 fg/reaction (Figure S7b), which was slightly higher in sensitivity as compared with the results obtained from RPA assays. Based on these results, we concluded that the wearable soft-contact RPA microdevice could be energetically applied for rapid detection of SARS-CoV-2 in a simpler manner and low-resource settings.

### 3.6. On-Site Detection of Amplicons Using SYBR Green I-Based Fluorescence Signal

**Figure 7:**
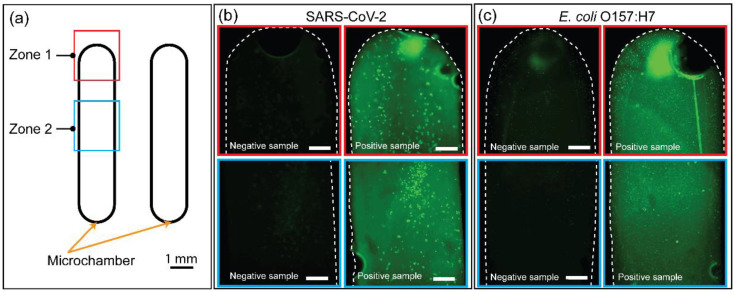
On-site detection of amplicons using the wearable RPA device. (**a**) Schematic illustration of the microdevice indicating two zones where the fluorescence images were captured. (**b**) Fluorescence signals of amplicons from DNA plasmid SARS-CoV-2 inside the PDMS microdevice. (**c**) Fluorescence signals of amplicons from *E. coli* O157:H7 inside the PDMS microdevice. Scale bars in (**b**,**c**) represent 200 μm.

In addition, to shorten the detection time of amplicons, SYBR Green I was added to the RPA sample after 23 min of incubation for the rapid detection-based fluorescence method. It is worth noting that SYBR Green I was only added after the microdevice was separated from the human body owing to a safety concern. Fluorescence images of two zones of the microchambers were obtained from the fluorescence images of two target genes (203 bp from plasmid SARS-CoV-2 and 210 bp from *E. coli* O157:H7), as shown in Figure 7a. The RPA results successfully amplified two gene fragments using a wearable PDMS microdevice, as shown in the gel image (Figure S8). Figure 7b,c show the fluorescence images of on-chip amplification of the 203 bp target band (plasmid SARS-CoV-2) and 210 bp target band (*E. coli* O157:H7) with SYBR Green I added after RPA assays. Based on these results, the strong green fluorescence signals of SYBR Green I intercalated with RPA amplicons (positive sample) of the on-chamber amplifications were significantly different from those of the negative sample, indicating consistent results between fluorescence measurements and gel electrophoresis, as shown in Figure S8. It is also noticed that the RPA reagents may contain some components that can strongly react with SYBR Green I, resulting in some high-intensity dots in the fluorescence images, as shown in Figure 7b,c. Thus, the successful on-site detection of RPA amplicons via fluorescence signals demonstrates the application potential of the wearable PDMS microdevice in resource-limited settings with high DNA amplification efficiency and rapid detection of amplicons.

## 4. Conclusions

In summary, we fabricated a wearable RPA microdevice utilizing PDMS substrates that can perform DNA amplification via human body heat. To produce soft-contact PDMS, the PDMS substrate was developed with high flexibility and adhesion by reducing the amount of curing agent in the mixing ratio of PDMS, without changing the PDMS properties. As a result, the mixing ratio of the 10:0.4 (*w*/*w*) PDMS substrate not only improved contact between the wearable microdevice and human skin but was also able to realize PDMS–PDMS bonding without surface modification. As compared with other wearable RPA devices which use additional support for holding the device such as a wristband, the current device offers wide ranges in size forming highly stable and conformal contact on human skin. Thus, the wearable microdevice was successfully employed to amplify two target genes for the detection of *E. coli* O157:H7 from gDNA and COVID-19 from DNA plasmid SARS-CoV-2 using human body heat with high sensitivity and specificity. By incorporating a sample preparation step, the current platform can be more suitable for POCT. In addition, the sensitivity of the current platform can be further improved by being integrated with smartphone-based detection, making the device more affordable and accessible in resource-limited settings.

## Data Availability

The data presented in this study are available on request from the corresponding author.

## Supplementary Materials

The following supporting information can be downloaded at: https://www.mdpi.com/article/10.3390/bios12020072/s1, Figure S1: (a) The key ingredients of RPA assays include DNA template, primers, nucleotides, recombinase, DNA polymerase, and single-strand DNA binding protein. (b) DNA amplification using the RPA technique. (i) Recombinase-primer complexes scan DNA for complementary sequences. Recombinase inserts primers via strand exchange. Single-strand DNA binding protein (SSB) binds to displaced strands, stabilizing the bound primer. (ii,iii) The primer was extended to the new strand by DNA polymerase. The parental strand is displaced, and elongation continues. (iv) Two copies of DNA are formed. The drawings were modified from [33]; Figure S2: (a) Photographs showing the wearable PDMS microdevice attached on the forearm. (b) Photographs showing 10:0.4 (*w/w*) mixing ratio of PDMS and pre-polymer demonstrating highly skin-compatible mechanical and soft-contact performance. (c) Results showing the leak test observed over 96 h; Figure S3: The 210 bp target gene from *E. coli* O157:H7 was successfully amplified based on RPA technique using thermal cycler with temperature ranging from 40 to 30 °C. Lane 1 is a negative control. Lanes 2–7 are RPA products obtained from reactions performed from 40 to 30 °C at 2 °C intervals. (b) The 210 bp target gene from *E. coli* O157:H7 was successfully amplified using RPA technique and human body heat with three different volunteers. Lane 1 is a negative control. Lanes 2–4 are RPA products obtained from three different volunteers. Lane M is 100 bp ladder; Figure S4: Illustration showing four positions for attaching tubes for RPA (P1—chest; P2—forearm; P3—wrist; and P4—fist) and real photographs of the tubes for RPA adhered on a volunteer’s skin at four designated positions using tape; Figure S5: Temperature variations measured on the wearable RPA microdevice at four positions, P1—chest, P2—forearm, P3—wrist, and P4—fist. Each temperature was measured ten times using a non-contact precision infrared thermometer; Figure S6: Temperature measurement. (a) Graphs showing the temperature variations measured on the wearable RPA microdevice at four positions, P1—chest, P2—forearm, P3—wrist, and P4—fist using an IR camera. (b) IR camera images showing the temperatures measured at P3—wrist and P4—fist for 60 min. Each temperature was measure ten times and averaged; Figure S7: Results of agarose gel electrophoresis showing the sensitivity using tenfold serial dilutions of plasmid DNA from SARS-CoV-2. (a) On-tube RPA results. (b) On-tube PCR results. Lane M is the 100 bp DNA ladder. Lanes 1–8 show the amplification results when input plasmid DNA concentrations were 0.06 fg to 60 pg/sample, respectively. All the experiments were repeated three times.; Figure S8: Agarose gel electrophoresis images of 203 bp (SARS-CoV-2) and 210 bp (*E. coli* O157:H7) target genes obtained using the wearable PDMS microdevice and human body heat. Lane M is the 100 bp DNA ladder. Lanes 1 and 2 are the negative and positive samples of SARS-CoV-2, respectively. Lanes 3 and 4 are the negative and positive samples of *E. coli* O157:H7, respectively.
