# Supplementary material for: Fabrication of Wearable PDMS Device for Rapid Detection of Nucleic Acids via Recombinase Polymerase Amplification Operated by Human Body Heat
Source: Biosensors (Basel). 2022 Jan 27;12(2):72. doi: 10.3390/bios12020072 (PMC8869297; doi:10.3390/bios12020072)
Supplement: Supplementary file 1 [file biosensors-12-00072-s001.zip › biosensors-1565219-supplementary.pdf]

biosensors

Supplementary Materials

Fabrication of Wearable PDMS Device for Rapid Detection of Nucleic Acids via Recombinase Polymerase Amplification Operated by Human Body Heat

Kieu The Loan Trinh [1] and Nae Yoon Lee [2,*]

[1] Department of Industrial Environmental Engineering, Gachon University, 1342 Seongnam-daero, Sujeong-gu, Seongnam-si, Gyeonggi-do, 13120, Korea; tktloan@gmail.com
[2] Department of BioNano Technology, Gachon University, 1342 Seongnam-daero, Sujeong-gu, Seongnam-si, Gyeonggi-do, 13120, Korea.; nylee@gachon.ac.kr
* Correspondence: nylee@gachon.ac.kr

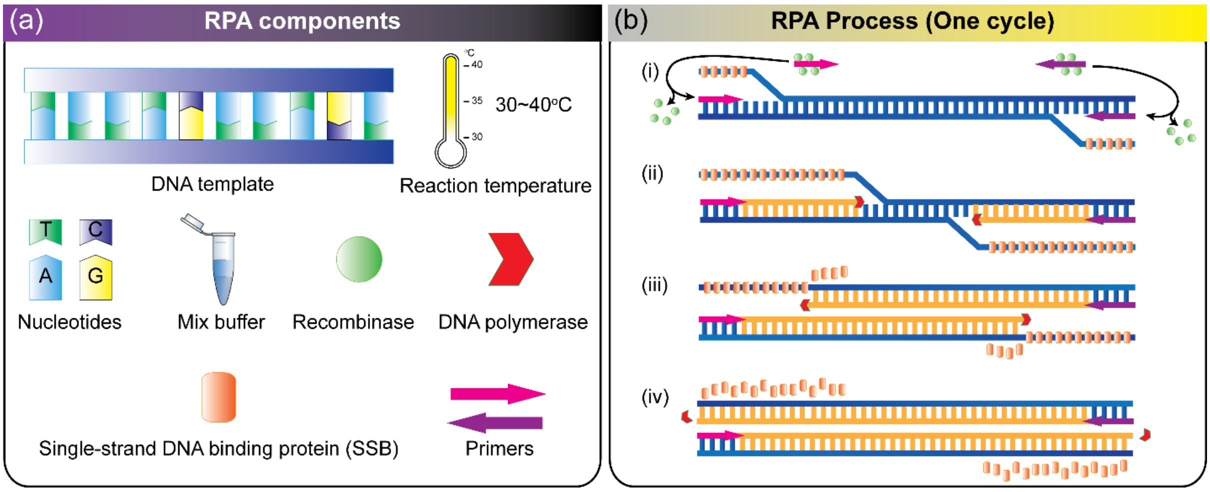

Figure S1. (**a**) The key ingredients of RPA assays include DNA template, primers, nucleotides, recombinase, DNA polymerase, and single-strand DNA binding protein (SSB). (**b**) DNA amplification using the RPA technique. (i) Recombinase-primer complexes scan DNA for complementary sequences. Recombinase inserts primers via strand exchange. SSB binds to the displaced strands, stabilizing the bound primers. (ii-iii) The primers were extended to the new strands by DNA polymerase. The parental strand is displaced and elongation continues. (iv) Two copies of DNA are formed. The drawings were modified from [33].

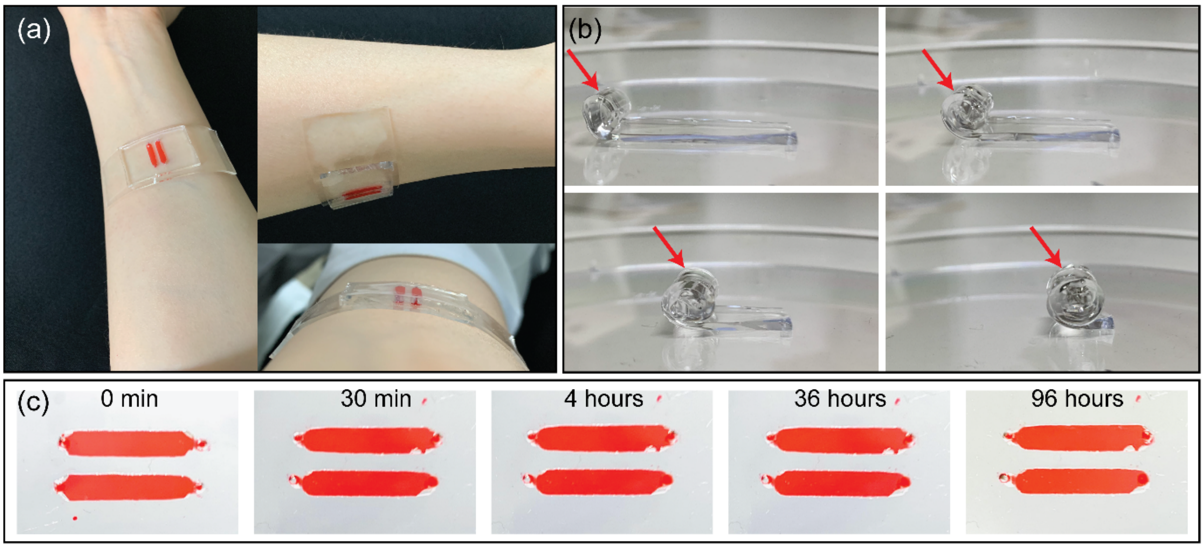

Figure S2. (**a**) Photographs showing the wearable PDMS microdevice attached on the forearm. (**b**) Photographs showing 10:0.4 (w/w) mixing ratio of PDMS and prepolymer demonstrating highly skin-compatible mechanical and soft-contact performance. (**c**) Results showing the leak test observed over 96 h.

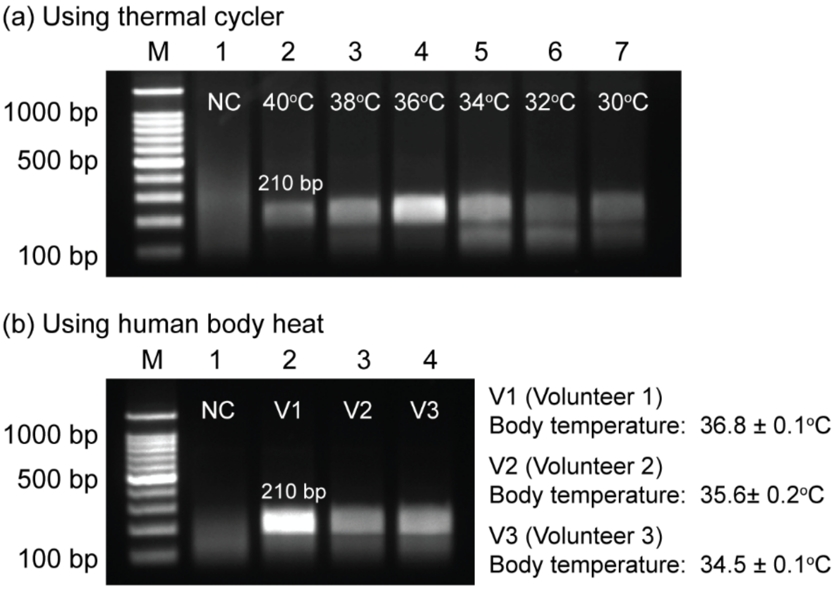

Figure S3. (**a**) The 210 bp target gene from *E. coli* O157:H7 was successfully amplified based on RPA technique using thermal cycler with temperature ranging from 40 to 30°C. Lane 1 is negative control. Lanes 2-7 are RPA products obtained from reactions performed from 40 to 30°C at 2°C intervals. (**b**) The 210 bp target genes from *E. coli* O157:H7 were successfully amplified using RPA technique and human body heat with three volunteers. Lane 1 is negative control. Lanes 2-4 are RPA products obtained from three volunteers. Lane M is 100 bp ladder.

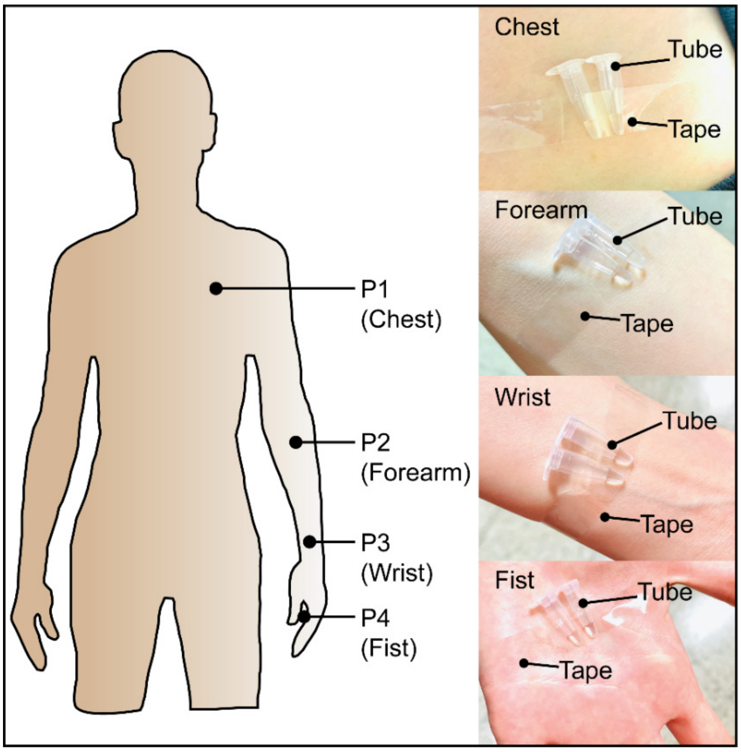

Figure S4. Illustration showing four positions for attaching tubes for RPA (P1-chest, P2-forearm, P3-wrist, and P4-fist) and real photographs of the tubes used for RPA reaction adhered on volunteer's skin at aforementioned four designated positions using tape.

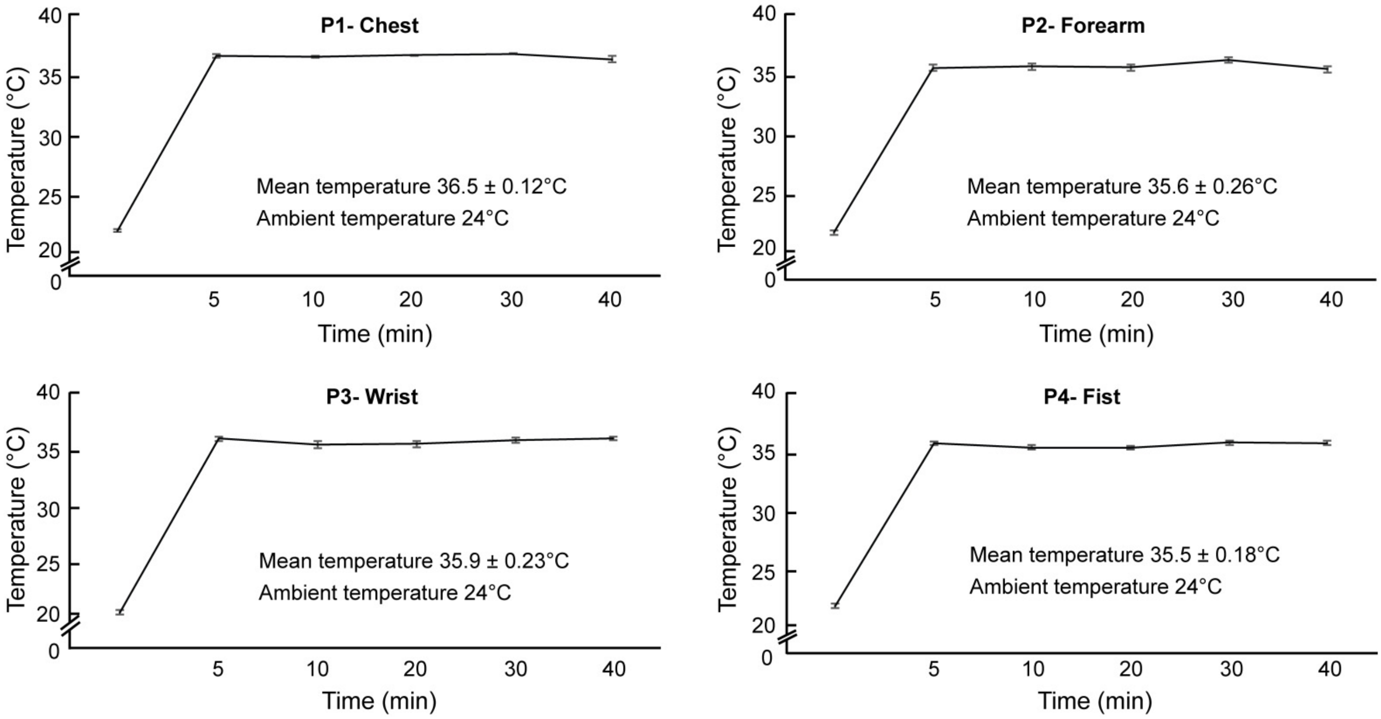

Figure S5. Temperature variations measured on the wearable RPA microdevice placed at four positions – P1-chest, P2-forearm, P3-wrist, and P4-fist. Each temperature was measured ten times using a non-contact precision infrared thermometer.

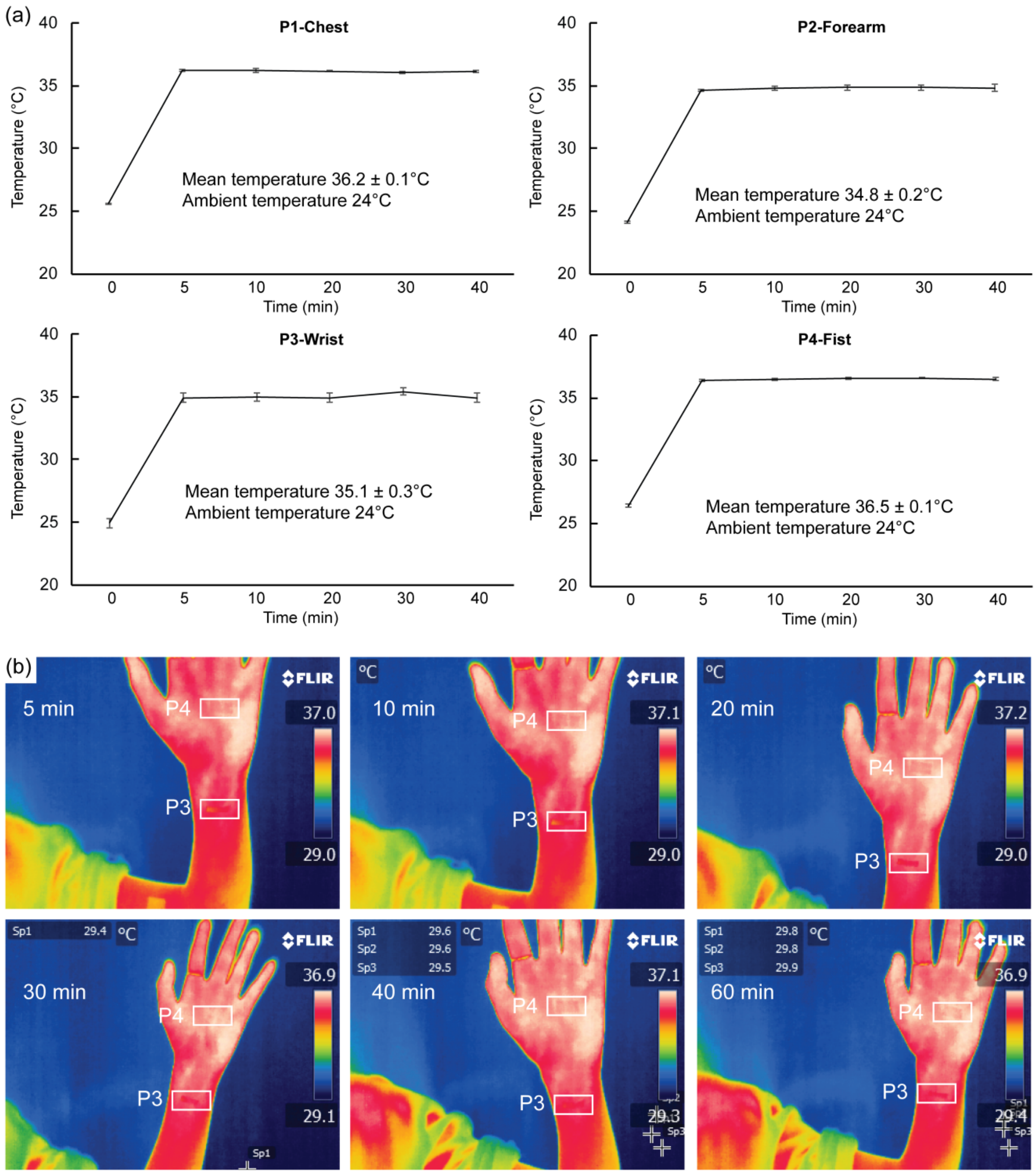

Figure S6. Temperature measurement. (**a**) Graphs showing the temperature variations measured on the wearable RPA microdevice at four positions using IR camera; P1-chest, P2- forearm, P3-wrist, and P4-fist. (**b**) IR camera images showing the temperatures measured at P3-wrist and P4-fist for 60 min. Each temperature was measure ten times and averaged.

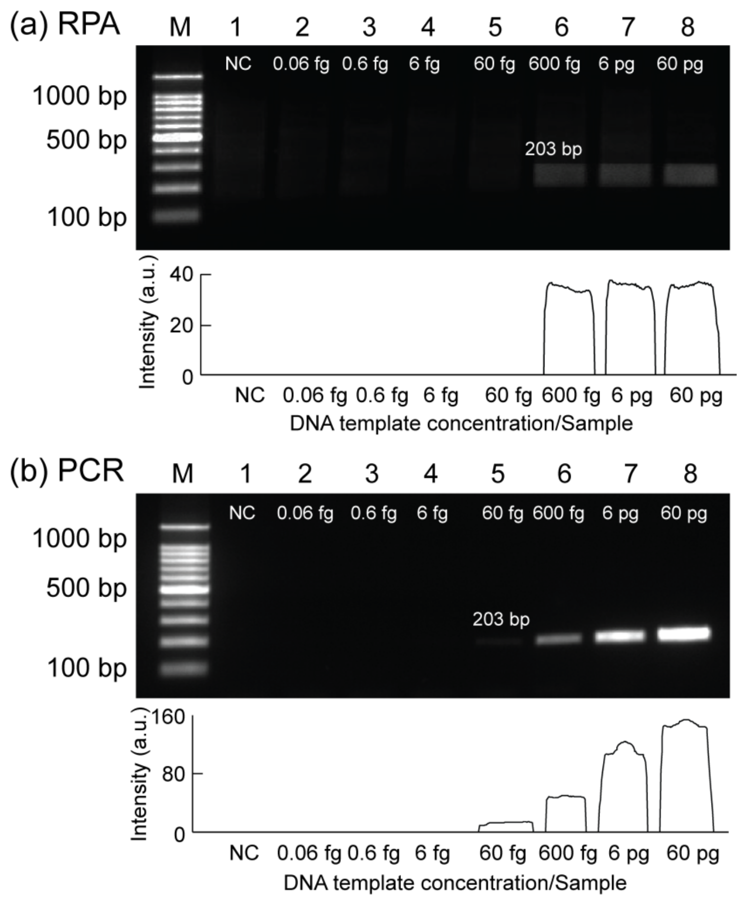

Figure S7. Results of agarose gel electrophoresis showing the sensitivity using ten-fold serial dilutions of plasmid DNA from SARS-CoV-2. (**a**) On-tube RPA results. (**b**) On-tube PCR results. Lane M is 100 bp DNA ladder. Lanes 1-8 show the amplification results when input plasmid DNA concentrations were 0.06 fg to 60 pg/sample, respectively. All the experiments were repeated three times.

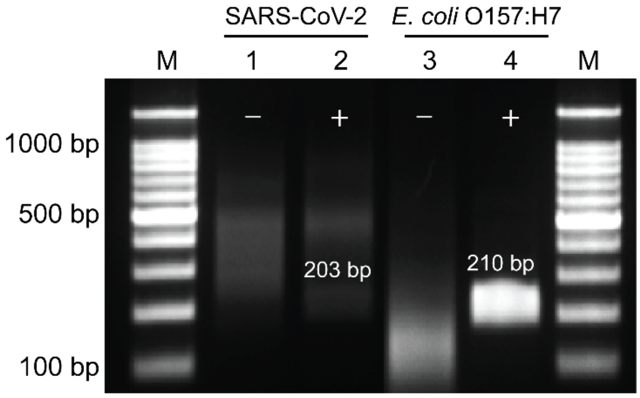

Figure S8. The image of agarose gel electrophoresis of 203 bp (SARS-CoV-2) and 210 bp (*E. coli* O157:H7) target genes obtained using the wearable PDMS microdevice and human body heat. Lane M is 100 bp DNA ladder. Lanes 1-2 are the negative and positive samples of SARS-CoV-2, respectively. Lanes 3-4 are the negative and positive samples of *E. coli* O157:H7, respectively.
